# Stent-Assisted Double Microcatheter Coil Embolization for an Inferior Pancreaticoduodenal Artery Aneurysm after Multiple Stent-Graft Procedures: A Case Report

**DOI:** 10.3400/avd.cr.26-00021

**Published:** 2026-04-24

**Authors:** Yoshiki Yamasaki, Hiroshi Nishimaki, Tsuyoshi Kono, Masahiro Tomita, Satoshi Kinebuchi, Daijun Tomimoto, Masahide Komagamine, Kan Nawata

**Affiliations:** Department of Cardiovascular Surgery, St. Marianna University School of Medicine, Kawasaki, Kanagawa, Japan

**Keywords:** inferior pancreaticoduodenal artery aneurysms, double microcatheter technique, celiac artery embolization

## Abstract

Inferior pancreaticoduodenal artery aneurysms (IPDAAs) are rare visceral aneurysms that may rupture irrespective of size. Here, we report an IPDAA that progressively enlarged after celiac artery (CA) embolization during aortic stent–graft repair. An 84-year-old female developed a wide-necked saccular IPDAA that expanded from 2.5 to 15 mm over 10 years after CA embolization. Stent-assisted coil embolization using the double microcatheter technique was performed to preserve superior mesenteric artery-to-CA collateral flow, achieving a packing density of 31%–45%. This case highlights the need for surveillance after CA embolization and supports the feasibility and effectiveness of this technique in select cases.

## Introduction

Visceral artery aneurysms (VAAs) are rare, accounting for approximately 0.1%–2% of all aneurysms; however, reported cases have increased with advances in imaging modalities.^1)^ Inferior pancreaticoduodenal artery aneurysms (IPDAAs) represent 2%–10% of all VAAs and are characterized by rupture risk independent of aneurysm size, with a reported rupture-related mortality rate of 21%–26%.^2)^ The mechanisms underlying IPDAAs include increased collateral flow through the pancreaticoduodenal arcade due to celiac artery (CA) stenosis or occlusion, or superior mesenteric artery (SMA) stenosis or dissection.^3)^ CA stenosis has been reported in 50%–80% of patients with PDAAs.^2,3)^

However, aneurysm formation or enlargement after intentional CA embolization is rarely reported, and its hemodynamic implications remain unclear. In this report, we describe a rare case of progressive IPDAA enlargement after CA embolization performed during endovascular aortic repair, which was successfully treated using a stent-assisted double microcatheter coil embolization technique.

## Case Report

An 84-year-old female (height, 150 cm; weight, 44 kg; body mass index, 19.6 kg/m^2^) had undergone ascending aortic replacement for Stanford type A acute aortic dissection 23 years previously. Over the next 13 years, she developed progressive dissecting thoracic and abdominal aortic aneurysms, for which thoracic endovascular aortic repair and endovascular aneurysm repair were performed. Concomitant CA embolization was performed to prevent type II endoleak.

Her medical history included hypertension, dyslipidemia, severe aortic regurgitation associated with a sinus of Valsalva aneurysm, atrial fibrillation complicated by heart failure, and a previous right vitreous hemorrhage. She was taking a direct oral anticoagulant (DOAC) for atrial fibrillation.

**Fig. 1:**
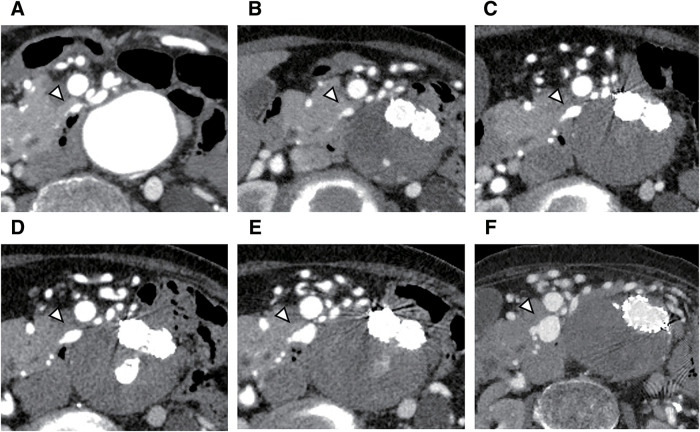
Serial computed tomography images demonstrating progressive enlargement of the (IPDAA, white arrowheads). (**A**) Before CA embolization, the IPDAA measured 2.5 mm. (**B**) Immediately after CA embolization, the aneurysm size remained unchanged (2.5 mm). (**C**) At 8 months post-embolization, the aneurysm enlarged to 5.0 mm. (**D**) At 2 years, the aneurysm enlarged to 6.5 mm. (**E**) At 5 years, the aneurysm size increased to 8.9 mm. (**F**) At 10 years, the aneurysm markedly enlarged to 15.0 mm. IPDAA: inferior pancreaticoduodenal artery aneurysm; CA: celiac artery

Before CA embolization, the maximum diameter of the inferior pancreaticoduodenal artery (IPDA) was 2.5 mm (**Fig. 1A**). The diameter remained unchanged immediately after CA embolization (**Fig. 1B**). During follow-up, serial imaging demonstrated gradual enlargement (5.0 mm at 8 months, 6.5 mm at 2 years, and 8.9 mm at 5 years) (**Fig. 1C**–**1E**), ultimately reaching a maximum diameter of 15 mm 10 years after CA embolization (**Fig. 1F**).

Computed tomography (CT) revealed a true saccular aneurysm measuring 13.6 × 13.9 × 15.2 mm (volume, 1504 mm^3^). The aneurysm had a wide neck with a neck width of 8.9 mm and a neck-to-dome (N/D) ratio of 1.7.

The distance between the aneurysm and the bifurcation of the anterior and posterior inferior pancreaticoduodenal arteries (AIPDA/PIPDA) was 3.2 mm, which was considered insufficient to secure an adequate landing zone for covered stent placement. Preservation of collateral flow from the SMA to the CA also precluded the use of an isolation technique. The 5.3 × 5.5-mm diameter of the parent artery at the aneurysmal segment was considered when selecting the stent size.

Stent-assisted coil embolization using a SMART stent (Cordis, Miami Lakes, FL, USA) was planned. To achieve high packing density, 2 microcatheters were positioned within the aneurysm.

**Fig. 2:**
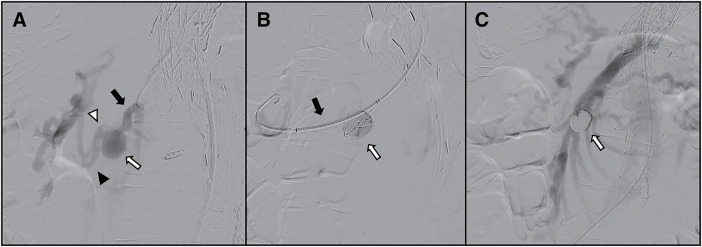
Intraoperative angiographic findings during stent-assisted coil embolization. (**A**) Selective angiography of the IPDA. A true saccular IPDA aneurysm (IPDAA) measuring 13.6 × 13.9 × 15.2 mm (volume, 1504 mm3) is shown (white arrow). The parent artery measured 5.3 × 5.5 mm in diameter (black arrow). The anterior IPDA (AIPDA; white arrowhead) and posterior IPDA (PIPDA; black arrowhead) are also visualized. (**B**) Deployment of a 6 × 40-mm SMART bare-metal stent from the IPDA to the AIPDA (black arrow). Two microcatheters are positioned within the aneurysm sac for coil embolization (white arrow). (**C**) Final angiography demonstrating complete exclusion of the aneurysm after dense coil packing (white arrow) with preservation of the distal arterial flow. IPDA: inferior pancreaticoduodenal artery

The procedure was performed under general anesthesia in a hybrid operating room. After systemic heparinization with intravenous heparin to maintain an activated clotting time (ACT) >200 s, a 12-Fr DrySeal sheath (W. L. Gore & Associates, Flagstaff, AZ, USA) was inserted through the right femoral artery, followed by placement and anteflexion of an Agilis NxT Steerable Introducer (Abbott, St. Paul, MN, USA). Access to the SMA–IPDA was achieved using an Impress Bernstein catheter (Merit Medical Systems, South Jordan, UT, USA), and the IPDA aneurysm and the surrounding vascular anatomy were confirmed (**Fig. 2A**).

After selective catheterization of the AIPDA, a 6 × 40-mm SMART stent was deployed across the aneurysmal neck. A coiling support catheter (Medico’s Hirata, Osaka, Japan) and an Excelsior 1018 microcatheter (Stryker Neurovascular, Cork, Ireland) were advanced into the aneurysm through the left femoral approach (**Fig. 2B**). After stent deployment, coil embolization was performed using 2 microcatheters—a combination of Target XL and XXL detachable coils (Stryker Neurovascular) and AZUR18 hydrogel-coated coils (Terumo, Somerset, NJ, USA)—to achieve dense intra-aneurysmal packing (**Fig. 2C**).

The final packing density was 31%–45%. After confirming the absence of complications, anticoagulation therapy was resumed on postoperative day 1. Distal perfusion was preserved, and the patient was discharged on postoperative day 4.

## Discussion

Because PDAAs are known to rupture irrespective of aneurysm size, the Society for Vascular Surgery guidelines recommend treatment regardless of diameter.^4)^ Reported etiologies include CA stenosis or occlusion (e.g., median arcuate ligament syndrome [MALS]), SMA origin stenosis or dissection, segmental arterial mediolysis, vasculitis, pancreatitis-related pseudoaneurysm, trauma, iatrogenic injury, and infection.^5)^ Among these, CA stenosis or occlusion is implicated in 50%–80% of PDAAs,^2,3)^ leading to hemodynamic stress through compensatory flow augmentation in the SMA–PDA arcade.^3)^ In the present case, the IPDA enlarged from 2.5 to 15 mm over 10 years after CA embolization, strongly suggesting that altered collateral flow contributed to aneurysm progression.

Surgery was performed after the aneurysm had enlarged to 15 mm. The aneurysm had enlarged to 8.9 mm within the first 5 years after CA embolization, and surgical treatment had been repeatedly recommended. However, the patient strongly preferred continued observation, and so conservative follow-up was maintained. Although earlier intervention might have been preferable, the timing of treatment was influenced by the patient’s strong preference for continued observation. The patient was subsequently followed up at another institution and was lost to our follow-up. When she returned to our hospital 10 years after CA embolization, the aneurysm had enlarged to 15 mm, and intervention was planned after explaining the potential risk of rupture.

In patients with PDAA associated with CA stenosis or occlusion, management of the underlying inflow lesion remains controversial. Additional treatment of the CA, such as surgical decompression in MALS or revascularization, may be considered in selected patients, particularly in younger individuals with long life expectancy, multiple aneurysms, high-flow collateral circulation, symptomatic MALS, or recurrent aneurysm-related findings. In contrast, strict imaging surveillance may be reasonable in elderly or high-risk patients with a single treated lesion and stable findings after aneurysm treatment. Therefore, treatment strategies should be individualized based on patient characteristics and hemodynamic considerations.^4,6)^

According to current treatment guidelines for VAAs, endovascular therapy is generally considered the first-line treatment when anatomically feasible, except for renal artery aneurysms.^4)^ When endovascular treatment is not feasible, open surgical repair or hybrid approaches may be considered.

In patients with MALS, surgical treatment may include median arcuate ligament release to relieve CA compression. In addition, aneurysm resection combined with bypass reconstruction to major visceral arteries has been described. Hybrid strategies combining open and endovascular techniques have also been reported. For example, parent artery isolation can be performed endovascularly while distal perfusion is preserved through surgical bypass reconstruction.^6)^

In the present case, the patient was elderly and had multiple comorbidities, including heart failure, and was therefore considered unlikely to tolerate open abdominal surgery. Surgical resection of pancreaticoduodenal artery aneurysms is technically difficult because of their deep peripancreatic location. In addition, bypass reconstruction was considered challenging, as the patient had undergone multiple prior aortic stent–graft procedures, which were expected to result in severe periaortic adhesions and make aortic clamping or anastomosis difficult. Under these circumstances, long-term graft patency could not be assured, and graft occlusion might have resulted in serious consequences. Therefore, an endovascular strategy was selected because it was anatomically feasible and less invasive than open or hybrid repair.

When planning the endovascular treatment strategy, preservation of collateral flow to the CA territory was an important consideration. Because the CA territory was supplied via the IPDA, parent artery embolization was considered inappropriate. In addition, covered stent placement was deemed unsuitable because the distance from the aneurysm to the distal bifurcation of the AIPDA/PIPDA was only 3.2 mm, which was insufficient to secure an adequate landing zone.

Consequently, intra-aneurysmal coil packing was considered the only feasible treatment option. Although sac packing alone is generally discouraged for pseudoaneurysms because of the lack of a true arterial wall and the associated risk of rupture or recanalization,^5)^ no imaging features suggestive of pseudoaneurysm were observed in this case. These findings supported the diagnosis of a true saccular aneurysm, allowing the safe application of this treatment strategy.

The aneurysm had a broad neck (N/D ratio, 1.7), making simple coil packing risky because of potential coil prolapse. Stent-assisted coil embolization is advantageous for wide-necked aneurysms and lesions located near major bifurcations, with favorable outcomes reported in VAAs.^5)^ Furthermore, the double microcatheter technique enables more homogeneous and stable coil packing for broad-necked saccular aneurysms.^7)^

Packing density ≥24% has been suggested to be associated with reduced rates of coil compaction and recanalization.^8)^ In this context, the use of hydrogel-coated coils may have contributed to achieving sufficient packing density in the present case. Therefore, the packing density of 31%–45% achieved in this case is considered sufficient to minimize recurrence risk.

Although no established guidelines address antiplatelet therapy after visceral artery stenting, several reports recommend dual antiplatelet therapy for a short period followed by long-term single antiplatelet therapy to reduce the risk of in-stent restenosis.^9)^ However, in the present case, antiplatelet therapy was not administered because the patient was already receiving a DOAC and had a history of bleeding. Therefore, anticoagulation therapy was continued without the addition of antiplatelet agents.

Follow-up imaging with contrast-enhanced CT or magnetic resonance angiography is generally performed within 3–6 months after treatment and periodically thereafter to detect aneurysm reperfusion, coil compaction, or in-stent restenosis.^10)^

This case is notable because IPDAA enlargement occurred in a unique hemodynamic environment after intentional CA embolization, and reports of PDAA development or progression following CA embolization are scarce. Although causality cannot be definitively established from a single case, the present findings indicate that altered collateral hemodynamics after CA embolization may contribute to aneurysm progression and underscore the need for careful long-term surveillance in such patients. In particular, these findings highlight the importance of monitoring collateral pathways after intentional CA embolization during aortic stent–graft procedures.

## Conclusion

PDAAs may enlarge after CA embolization, requiring vigilant imaging surveillance. For wide-necked IPDAAs, stent-assisted coil embolization using the double microcatheter technique is a safe and effective option that can achieve high packing density.
